# Decoding the application of deep learning in neuroscience: a bibliometric analysis

**DOI:** 10.3389/fncom.2024.1402689

**Published:** 2024-10-04

**Authors:** Yin Li, Zilong Zhong

**Affiliations:** ^1^Nanyang Institute of Technology, Nanyang, China; ^2^Beijing Foreign Studies University, Beijing, China

**Keywords:** deep learning, neuroscience, bibliometric analysis, neural networks, computational models

## Abstract

The application of deep learning in neuroscience holds unprecedented potential for unraveling the complex dynamics of the brain. Our bibliometric analysis, spanning from 2012 to 2023, delves into the integration of deep learning in neuroscience, shedding light on the evolutionary trends and identifying pivotal research hotspots. Through the examination of 421 articles, this study unveils a significant growth in interdisciplinary research, marked by the burgeoning application of deep learning techniques in understanding neural mechanisms and addressing neurological disorders. Central to our findings is the critical role of classification algorithms, models, and neural networks in advancing neuroscience, highlighting their efficacy in interpreting complex neural data, simulating brain functions, and translating theoretical insights into practical diagnostics and therapeutic interventions. Additionally, our analysis delineates a thematic evolution, showcasing a shift from foundational methodologies toward more specialized and nuanced approaches, particularly in areas like EEG analysis and convolutional neural networks. This evolution reflects the field’s maturation and its adaptation to technological advancements. The study further emphasizes the importance of interdisciplinary collaborations and the adoption of cutting-edge technologies to foster innovation in decoding the cerebral code. The current study provides a strategic roadmap for future explorations, urging the scientific community toward areas ripe for breakthrough discoveries and practical applications. This analysis not only charts the past and present landscape of deep learning in neuroscience but also illuminates pathways for future research, underscoring the transformative impact of deep learning on our understanding of the brain.

## 1 Introduction

The integration of deep learning in neuroscience research represents a significant advancement in understanding the complex workings of the brain (Yin et al., 2022). This interdisciplinary approach combines the computational power of deep learning with the biological insights of neuroscience, promising to unravel many mysteries of neural functioning and brain disorders (Bart and Hegdé, 2019). Neuroscience is pivotal in understanding the structure and function of the nervous system, encompassing various aspects like cognitive processes, neural development, and brain disorders. It bridges multiple disciplines, contributing to fields as diverse as psychology, medicine, and computer science (Kasemsap, 2017). The insights gained from neuroscience are essential for developing treatments for neurological disorders, understanding human behavior, and even in creating more advanced computational models.

Deep learning offers a transformative potential for neuroscience (Saxe et al., 2021). It provides tools for analyzing complex, high-dimensional neuroimaging data, leading to improved diagnostic classifications and predictions in conditions like Alzheimer’s disease (Jo et al., 2019; Vallabhaneni et al., 2021). Moreover, deep learning models, inspired by neural networks in the brain, contribute to our understanding of cognitive processes and brain function (Botvinick et al., 2020). These models not only enhance our comprehension of neural mechanisms but also offer novel approaches in computational neuroscience, such as those found in the exploration of artificial general intelligence (AGI) inspired by brain-like intelligence mechanisms. Recent advancements in AGI have focused on mimicking the brain’s efficiency and adaptability, leading to significant breakthroughs in creating systems with high-level intelligence, high accuracy, high robustness, and low power consumption. Notably, studies on brain-inspired intelligence have introduced novel methodologies that stand out for their contribution to the field. For instance, the approach of utilizing the nonlinear information bottleneck (NIB) in spiking neural networks (SNNs) as presented in Yang and Chen (2023a) introduces an efficient way to process and transmit information by selectively filtering relevant neural spikes. This method enhances the performance of SNNs, making them more powerful and energy-efficient for tasks requiring complex information processing. Similarly, the study Yang and Chen (2023b) explores the use of high-order statistical dependencies in neural representations, significantly improving learning efficiency and robustness in SNNs. Furthermore, Yang et al. (2023a) demonstrates a novel learning algorithm that optimizes both robustness and energy efficiency in SNNs, showcasing the potential of these networks in real-world applications where power consumption is a critical factor. Yang et al. (2023b) explores the multi-scale learning capabilities of SNNs by mimicking the hybrid mechanisms of biological dendrites, offering insights into the versatility and adaptability of spike-based learning models. These pioneering studies mark a crucial step toward realizing brain-inspired AGI systems that can rival traditional artificial intelligence in efficiency and capability. Their focus on leveraging the unique properties of spiking neural networks to achieve breakthroughs in machine learning embodies the ongoing synergy between deep learning and neuroscience. By incorporating brain-like mechanisms, these approaches offer promising avenues for the development of AGI systems that are not only powerful and efficient but also robust and adaptable to a wide range of tasks and environments.

The exploration of deep learning in neuroscience has witnessed significant progress, yet a comprehensive bibliometric visual analysis examining this intersection remains scarce. This study addresses this gap by providing a macroscopic overview of the trends and hotspots in deep learning applications within neuroscience, offering a novel perspective on the evolution of this interdisciplinary field. There has been a mutual inspiration between deep learning and neuroscience, with each field advancing the other. Machine learning, particularly deep learning, has significantly contributed to neuroimaging and cognitive neuroscience, enabling sophisticated analysis of complex neural data (Helmstaedter, 2015). This study aims to fill the existing research gap by conducting a bibliometric analysis, identifying key trends and hotspots in the application of deep learning within neuroscience. The goal is to provide a comprehensive overview of the field’s trajectory and emerging focus areas. Utilizing Bibliometrix, a bibliometric software package in R, the study analyses 421 articles published between 2012 and 2023 (the first paper was published in 2012). This approach allows for a detailed visual analysis of the literature, revealing patterns and shifts in research focus over time. The study’s findings are expected to make significant contributions to the field, informing researchers of prevailing trends and potential future directions in the intersection of deep learning and neuroscience. It also aims to highlight areas that require further investigation, guiding subsequent research efforts.

## 2 Method

### 2.1 Data collection

**Figure 1:**
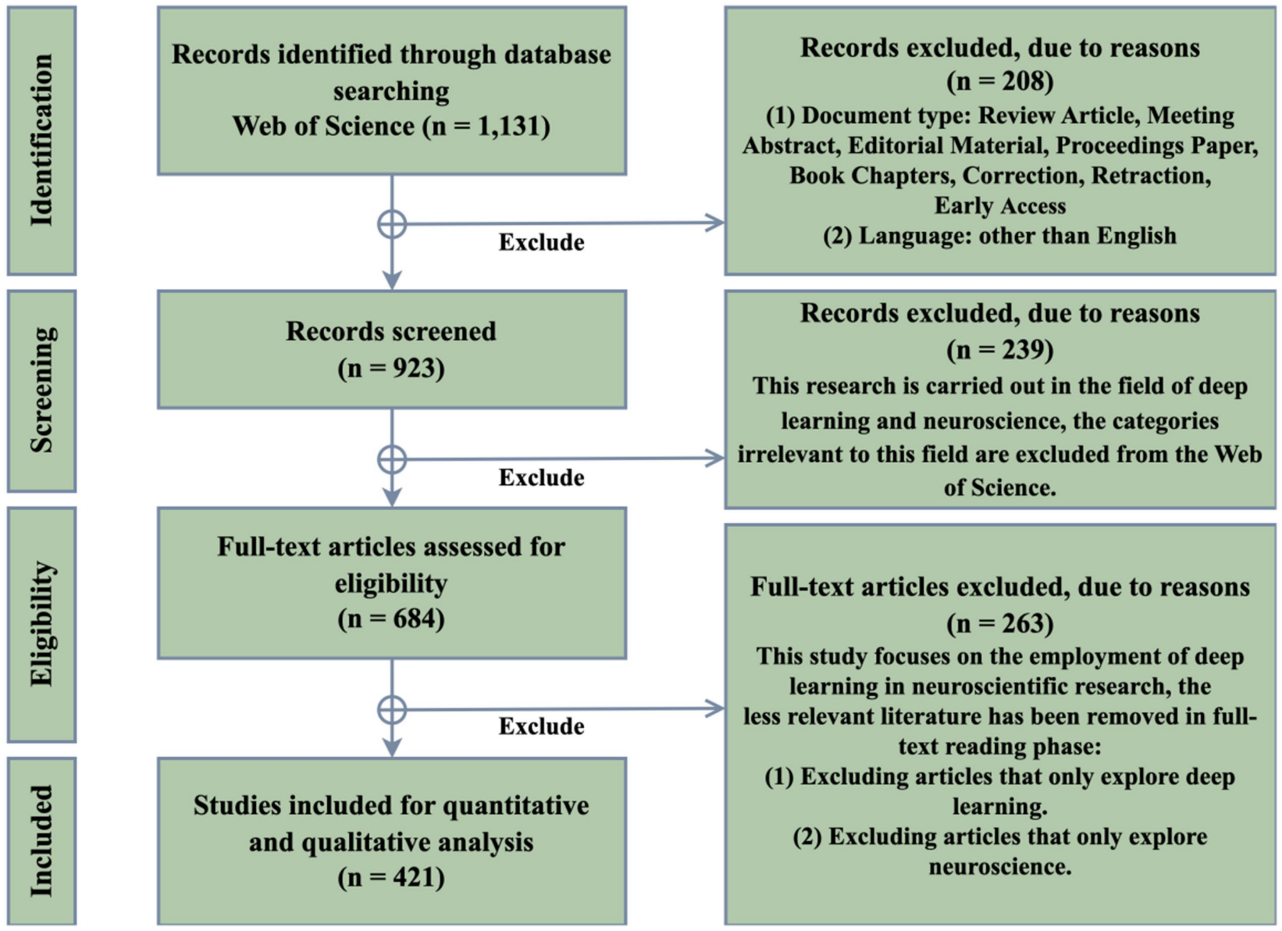
Workflow diagram of documents searching phase and the result of each stage in this study.

Bibliometric analysis is a statistical method for exploring and analyzing large volumes of scientific data. It is instrumental in understanding the evolution of specific fields and identifying emerging areas (Donthu et al., 2021). Bibliometric analysis is crucial for mapping the state of the art in a scientific field and identifying research gaps and trends (De Oliveira et al., 2019). The Web of Science (WoS) is a commonly used bibliographic database for bibliometric analysis. It is known for its stringent criteria compared to other databases, making it a reliable source for high-quality scientific information (Ellegaard and Wallin, 2015). The study collected data from the WoS Core Collection, focusing on papers published on deep learning for neuroscience from 2012 to 2023. The WoS Core Collection components included in this study are the Science Citation Index Expanded (SCI-Expanded), Social Sciences Citation Index (SSCI), Arts & Humanities Citation Index (A&HCI), and Emerging Sources Citation Index (ESCI). The search strategy employed was “Topic = (‘deep learning’ OR ‘artificial intelligence’ OR ‘artificial neural networks’) AND (‘neuroscience’ OR ‘brain science’).” This was complemented by specific inclusion criteria as outlined in Figure 1 of the study. A total of 421 records were gathered from 188 journals across 87 WoS categories. This extensive collection provides a comprehensive overview of the research landscape in the domain of deep learning for neuroscience.

### 2.2 Instrument

The instrument employed in this study is the Bibliometrix R-tool, a comprehensive tool designed for conducting science mapping and bibliometric analysis. Developed by Aria and Cuccurullo in 2017, Bibliometrix is an open-source tool programmed in R, offering flexibility and integration with other statistical R-packages. It supports a recommended workflow for bibliometric analyses, making it an ideal choice for exploring and analyzing large volumes of scientific data, especially in fields where empirical contributions are substantial and diverse (Aria and Cuccurullo, 2017). The purpose of this study is to employ Bibliometrix for conducting a bibliometric analysis of the scientific literature on deep learning in neuroscience. The objectives are to map the state of the art in this field, identify research gaps, and uncover trends and hotspots in the literature. The Bibliometrix R-tool will be instrumental in achieving these objectives by enabling the analysis of bibliographic data to visualize and quantify the scientific production, impact, and evolution of this research area.

### 2.3 Parameters

The bibliometric analyses conducted in this study utilized the Bibliometrix R-tool, tailored through specific parameter settings to enhance the relevancy and focus of the results. Annual publications analysis was performed without threshold adjustments to provide an unfiltered view of the research output over time. Keyword frequency analysis included keywords that appeared in at least 5% of the total publications, pinpointing the core concepts driving the field. For the thematic map analysis, keywords with a minimum occurrence of 5 and appearing in at least 5% of the publications were analyzed to visualize the connections between significant research themes robustly. The thematic evolution analysis employed a sliding time window, considering keywords appearing in at least 5% of publications during certain periods, which allowed for tracking the progression and shifts in thematic focus over time. Finally, the trend topics analysis identified rapidly emerging themes by selecting keywords that demonstrated a minimum 50% frequency increase in the certain years of the dataset. These parameter settings were chosen to ensure a detailed and insightful exploration of the expansive dataset, enabling a comprehensive understanding of the evolving dynamics within the domain of deep learning for neuroscience.

## 3 Results and discussion

### 3.1 Analysis of annual publications

**Figure 2:**
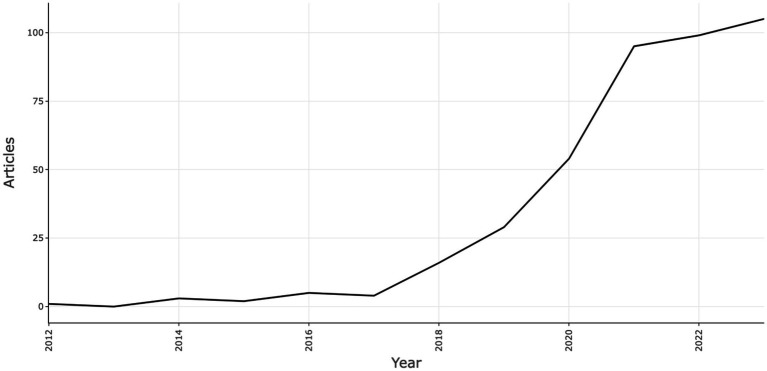
Annual publications of research on deep learning in neuroscience.

The trajectory of deep learning within neuroscience from 2012 to 2023 is marked by a significant increase in research output, as evidenced by bibliometric data (Figure 2). The period between 2012 and 2015, termed the foundational phase, saw an average of fewer than three publications per year, focusing on establishing core frameworks and potential deep learning applications in neuroscience. The subsequent years, particularly 2018 and 2019, witnessed a surge in publications to 16 and 29, respectively, highlighting early successful applications of deep learning in neurological studies, which catalyzed further interest and investment in this interdisciplinary area. The years 2020 to 2023 are characterized by rapid growth and maturation, with publications nearly doubling from 54 in 2020 to 95 in 2021, and then stabilizing at approximately 100 annually in the following years. This suggests a shift from exploratory to more in-depth research, targeting specific neurological challenges with refined deep learning techniques. The consistent publication rate indicates a matured field, with an established researcher and project base, a typical pattern for evolving research domains. In summary, the bibliometric analysis reveals a dynamic evolution from an emerging to a well-established field, highlighting deep learning’s critical impact on neuroscientific advancements. The recent focus on deeper, quality research suggests a promising direction toward significant discoveries in neuroscience.

### 3.2 Analysis of keyword frequency

**Figure 3:**
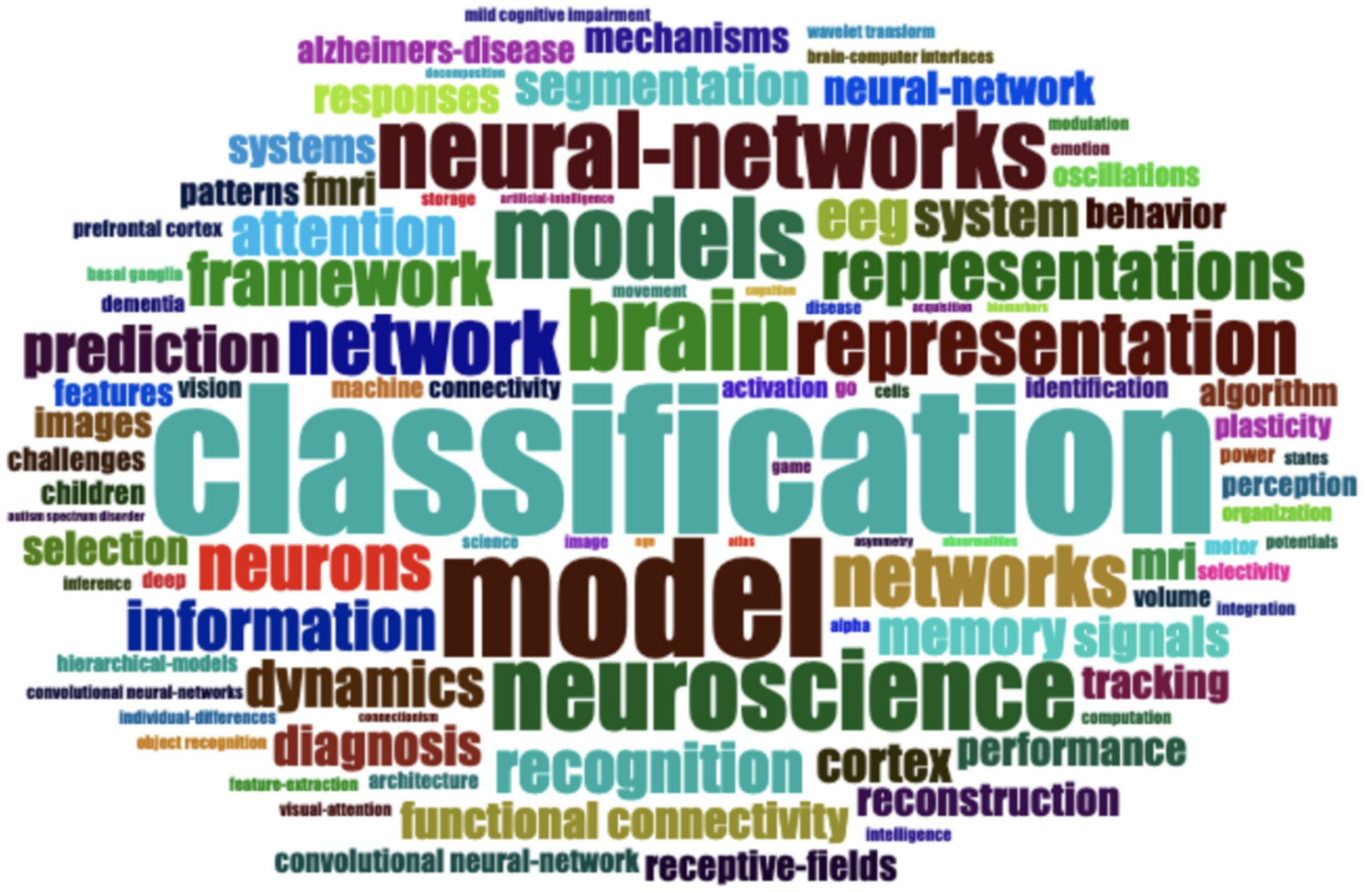
Word cloud based on keywords related to deep learning in neuroscience. This figure presents a word cloud visualization, where the size of each keyword reflects its frequency across the literature surveyed. This visualization highlights the most prominent terms and concepts in the field of deep learning applications within neuroscience.

The word cloud (Figure 3) generated from the keywords associated with deep learning and neuroscience research from 2012 to 2023 provides a vivid illustration of the field’s dynamic landscape. Central to this landscape is the concept of “classification,” appearing with the highest frequency. This term’s prominence underscores the pivotal role that classification algorithms play in interpreting complex neural data, from distinguishing between different states of brain activity to diagnosing neurological conditions. The terms “model” and “models” are also prevalent, indicating a substantial emphasis on the creation and refinement of computational models that simulate neural processes or predict outcomes. These models are not just theoretical constructs but serve as practical tools for understanding the intricate mechanisms of the brain. They are also instrumental in applying theoretical neuroscience findings to solve real-world problems, such as designing brain-computer interfaces or enhancing machine perception. “Brain,” “neuroscience,” and “cortex” point to the central focus of these studies: understanding the human brain’s function and structure. This encompasses a wide array of research objectives, from mapping the neural correlates of cognitive processes to advancing our understanding of the cortex’s role in diseases like Alzheimer’s. The frequent occurrence of “neural networks” and “network” signifies the close relationship between artificial neural network models in deep learning and the brain’s own neural networks. This mirroring between artificial and biological systems is a key area of exploration, as insights from one domain can often be translated into advancements in the other. “Representation” and “representations” refer to how information is encoded in the brain, a topic of immense interest for developing algorithms capable of mimicking or interpreting cognitive functions. Similarly, “framework” and “system” suggest the establishment of structured methodologies and comprehensive systems for research, indicating a maturation of the field’s investigative approaches. Terms like “neurons,” “functional connectivity,” “information,” “dynamics,” “memory,” “signals,” and “EEG” reflect the multi-faceted nature of neuroscience research, where studies range from the cellular level, examining individual neurons, to the systemic, exploring how different brain regions communicate and process information. The mention of “EEG” highlights the practical application of deep learning to interpret electroencephalographic data for various purposes, such as monitoring brain health or understanding cognitive states. Lastly, “prediction,” “diagnosis,” “segmentation,” and “signals” indicate a clinical application of deep learning, where predictive models are developed for early diagnosis and personalized treatment strategies, and image segmentation aids in the analysis of medical scans.

The word cloud encapsulates the interdisciplinary nature of this research domain. It underscores a concerted effort to leverage computational power to unravel the brain’s complexities, with the ultimate goal of advancing neuroscience through the lens of deep learning. This synergy is not just expanding our theoretical knowledge but also paving the way for innovative applications that could revolutionize the diagnosis and treatment of neurological disorders.

### 3.3 Analysis of thematic map

**Figure 4:**
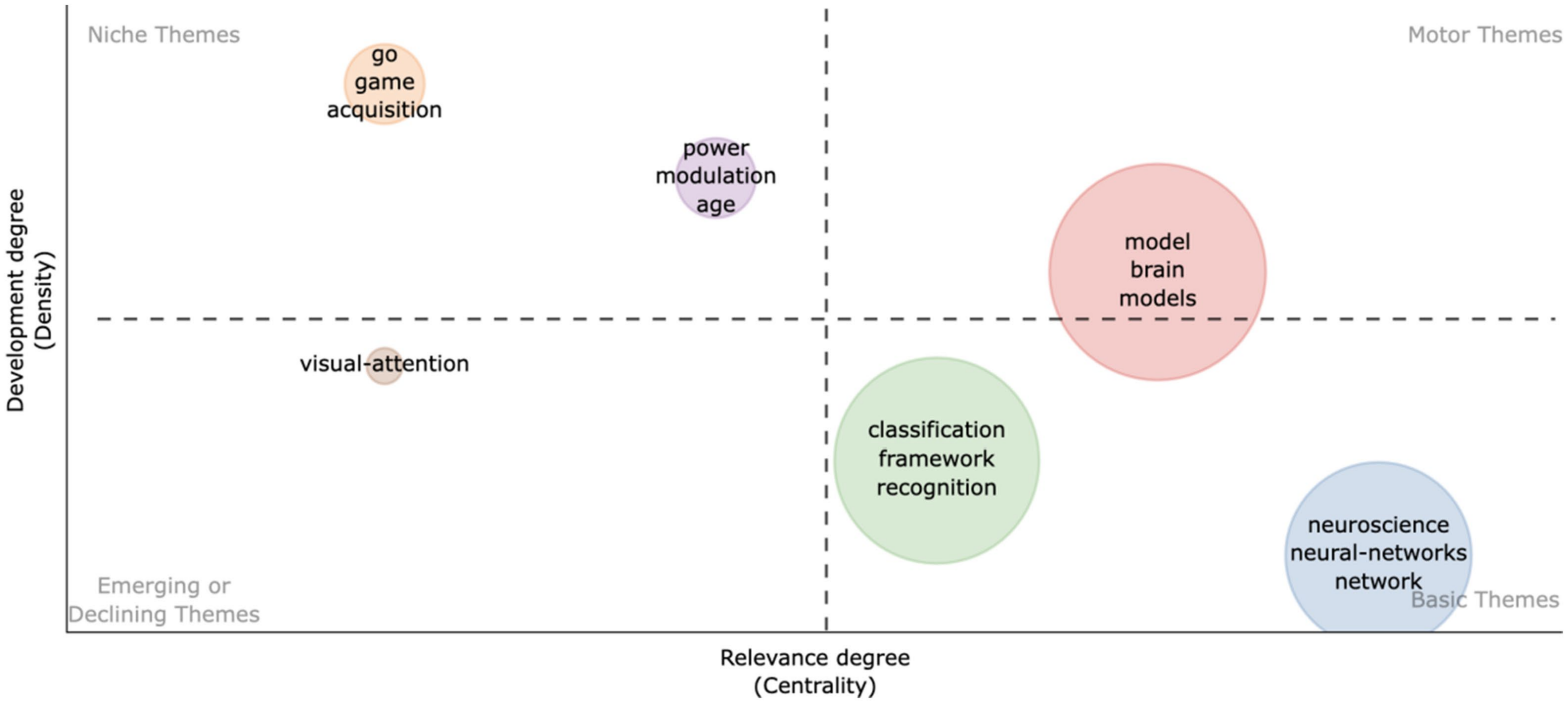
Thematic map of keywords in research on deep learning in neuroscience. This thematic map categorizes keywords into four quadrants reflecting their centrality and density in the literature: Motor themes (high centrality, high density), Niche themes (low centrality, high density), Emerging or Declining themes (low centrality, low density), and Basic themes (high centrality, low density). The size of the dots represents the occurrence frequency of each keyword.

The thematic map in Figure 4, generated using the Bibliometrix R-package, provides a structured overview of the deep learning themes in neuroscience by employing two distinct metrics: Density and Centrality. Density is calculated as the sum of the weights of the links connecting a theme to all other themes, normalized by the total number of themes. This metric reflects the internal strength and coherence of research within a theme, quantifying how well-developed and consolidated the topic is within the field. Centrality, on the other hand, is computed based on the degree centrality method. It measures the number of links that connect a theme to other themes, normalized by the maximum number of links that could possibly exist between this theme and others. This provides a measure of a theme’s influence and interaction with the broader research landscape, indicating its importance across disciplinary boundaries.

Key themes like “model,” “brain,” and “models” emerge as central, denoting both their mature development and foundational role in the field. These themes underscore the significance of computational models in simulating neural processes and the overarching focus on brain research (Linderman and Gershman, 2017; Whittington and Bogacz, 2019). Other themes such as “Go,” “game,” “acquisition,” “power,” “modulation,” and “age” are identified as niche areas with high internal development but limited external interactions, pointing to specialized or emerging research domains (Kovbasiuk et al., 2022; Edwards et al., 2023; Elsherif et al., 2023). “Visual-attention” is categorized as either emerging or declining, suggesting fluctuating research interest potentially revived by new technological advancements (Rees and Lavie, 2001; Obaidellah et al., 2018). Themes like “classification,” “framework,” “recognition,” “neuroscience,” “neural-networks,” and “network” occupy a quadrant indicative of fundamental but less developed areas, hinting at potential future research hotspots (Richards et al., 2019; Mehrer et al., 2020). This thematic map serves as a navigational tool for the intellectual structure of deep learning in neuroscience, offering insights into topic maturity and emerging research avenues, thereby guiding researchers in identifying developmental opportunities or areas needing attention.

### 3.4 Analysis of thematic evolution

**Figure 5:**
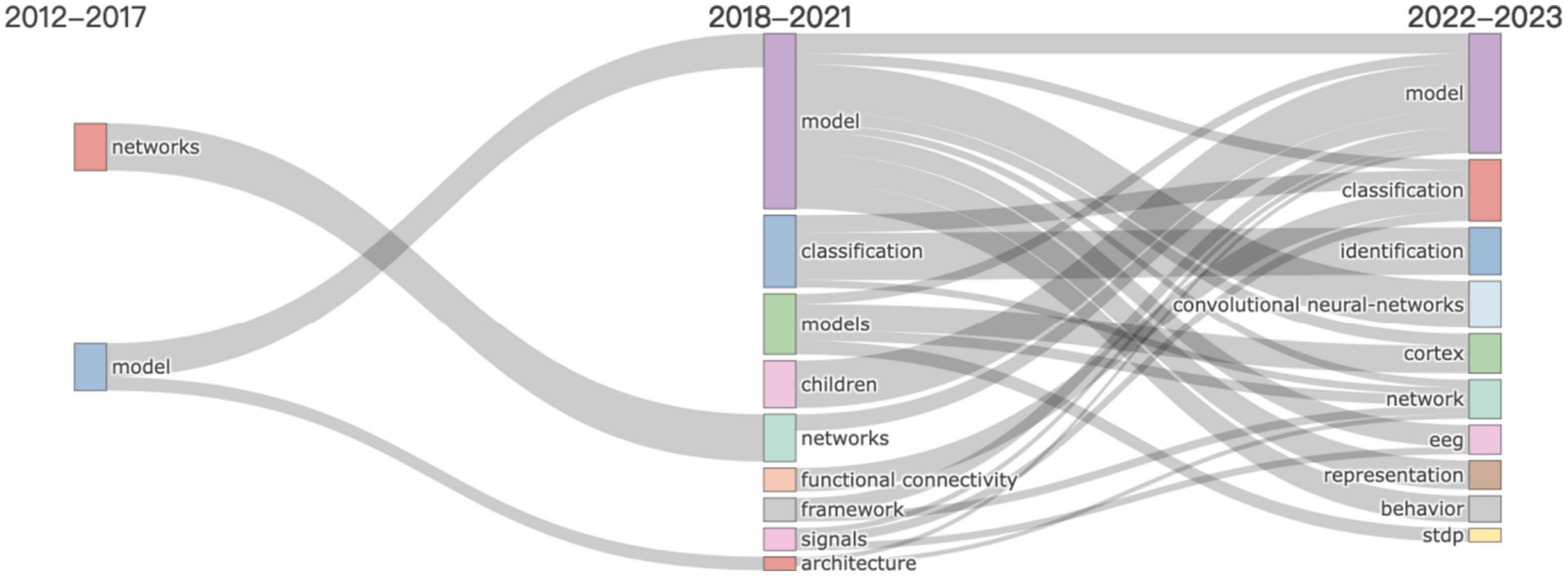
Thematic evolution of keywords in research on deep learning in neuroscience. This figure illustrates the progression of research themes over time, segmented into distinct time slices. Each line traces the evolution of a particular keyword through different periods, showing its development or decline in prominence within the field. The thickness of the lines indicates the volume of publications associated with each keyword during each period.

The thematic evolution map (Figure 5) is constructed using a combination of co-occurrence analysis and trend analysis techniques within the Bibliometrix R-package, enabling us to trace the dynamic shifts in focus and linkages among crucial concepts in deep learning and neuroscience from 2012 to 2023. This map is generated by first identifying key terms and their frequencies across different time slices within the dataset. Subsequently, a co-occurrence matrix is created for each period, mapping the frequency with which pairs of terms appear in the same publications. This allows for the visualization of connections and the prominence of themes over time.

Initially (2012–2017), emphasis was on foundational neural network models (“model” and “networks”), transitioning toward specialized architectures (“architecture”) by 2018–2021, indicating a move toward more nuanced neural network structures. The period also saw “classification” as a pivotal theme, emphasizing the importance of pattern interpretation in neuroscience data. The emergence of “children” in the discourse signals growing interest in applying deep learning to developmental neuroscience. Throughout 2018–2021, the consistent focus on “framework” and “functional connectivity” illustrates the ongoing effort to refine methods for examining brain connectivity. The term “signals” points to the rising significance of signal processing in neural data analysis during this time. The latest phase (2022–2023) underscores a continued interest in model-based approaches, with “convolutional neural-networks” indicating a focus on visual and spatial data analysis, crucial for brain imaging studies. The mention of “cortex” and “EEG” reflects an intensified focus on the cerebral cortex and EEG data’s role in informing deep learning models, while “behavior” suggests integrating behavioral data to develop comprehensive brain function models. The inclusion of “stdp” (spike-timing-dependent plasticity) indicates a growing interest in the mechanisms of neural circuit learning.

This evolution points to a field growing in sophistication, delving into specific areas while retaining core themes. The journey from broad modeling concepts to precise applications, such as EEG analysis and convolutional neural networks, mirrors the discipline’s maturation. The consistent presence of “classification” underscores the enduring challenge of deciphering complex neural data, central to marrying deep learning with neuroscience.

### 3.5 Analysis of trend topics

**Figure 6:**
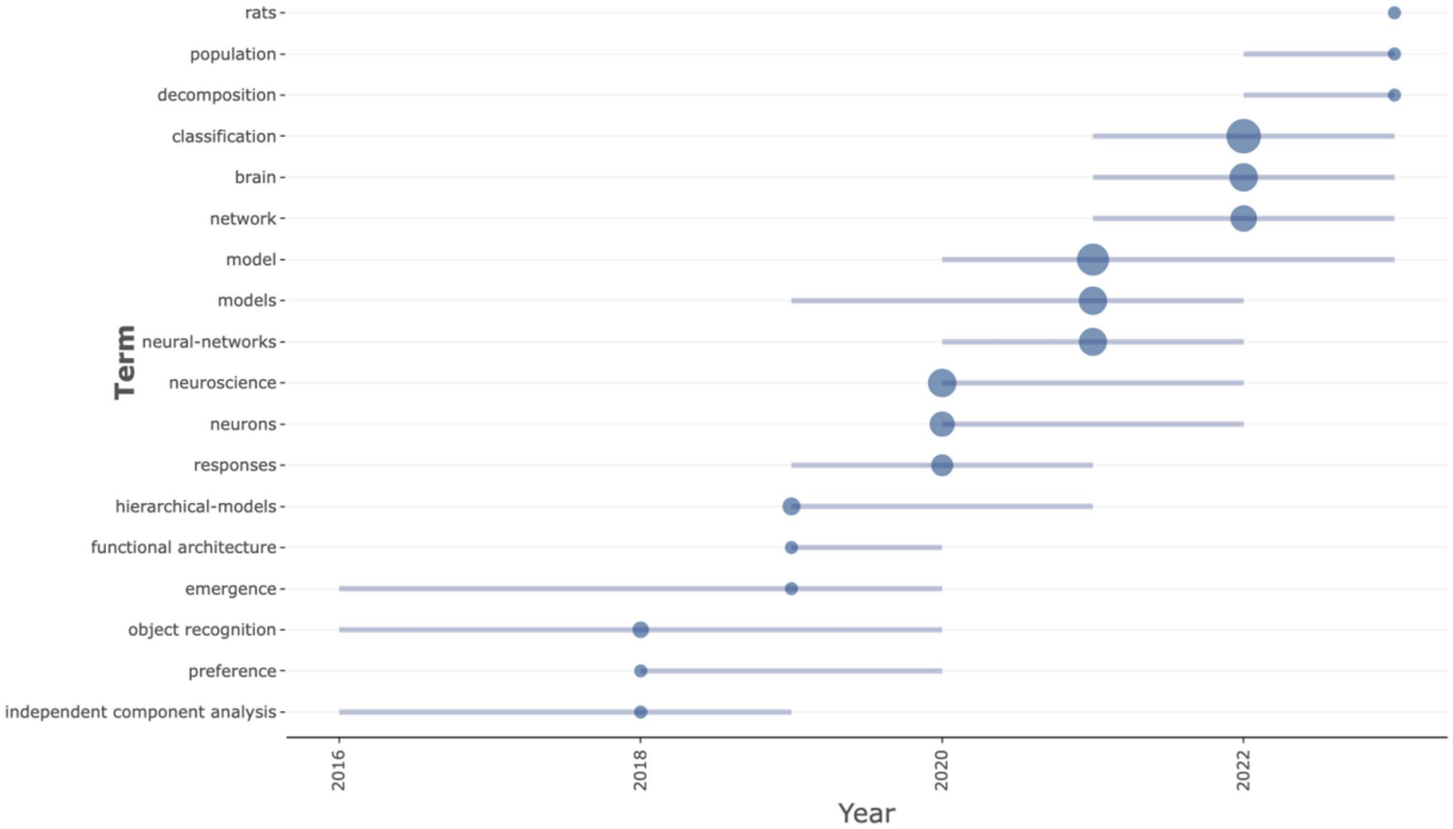
Trend topics by keywords of research on deep learning in neuroscience. This trend analysis plot displays the trajectory of major research topics over the years, using lines to represent the continuation of each topic based on keyword analysis. Each dot on the lines marks an annual data point, showing the relative prominence of topics in a given year.

The analysis of trend topics presents a chronological unfolding of key research themes in the domain of deep learning applied to neuroscience, as delineated by the frequency and temporal distribution of specific keywords from 2015 to 2023 (Figure 6). Initially, terms like “independent component analysis,” “object recognition,” and “emergence” indicated nascent interest in dissecting complex neural data and recognizing patterns. This period reflects a time when the field was actively exploring various computational techniques to unravel the brain’s complexities. “Hierarchical models” and “functional architecture,” peaking around 2019, point toward a developmental stride in understanding and modeling the layered structure of neural processing systems. Keywords such as “neural networks,” “neurons,” and “neuroscience” reaching their median mentions by 2020 and 2021 suggest a period of consolidation in the field, where the focus was on applying neural network models to neurological data and understanding neuron behavior. “Responses” gaining attention during this phase indicates increased interest in how the brain reacts to various stimuli and the implications for conditions such as neurodegenerative diseases or cognitive neuroscience. In the most recent phase, “classification” emerges as the predominant theme, dominating the research landscape from 2021 to 2023. This indicates a strong, ongoing focus on the categorization and interpretation of complex neural data, which is critical for advancements in diagnostics and therapeutic strategies. Similarly, “brain” and “network” ascending to their third quartile by 2023 reflect a sustained effort in understanding brain function and the intricate web of neural connections. A notable mention is the keywords “rats,” “population,” and “decomposition” reaching their first quartile in 2023, suggesting novel or renewed research interests. The use of “rats” signifies an increase in *in vivo* studies to test neural theories or the efficacy of neural network models. “Population” refer to population-level analyses in neuroscience, driven by large-scale neuroimaging studies or epidemiological approaches to neurological disorders. “Decomposition” is indicative of advanced analytical techniques being employed to break down complex neural signals or patterns.

Taken together, this trend analysis encapsulates the trajectory from exploratory computational methods to a deeper focus on classification and network analysis in recent years. The field appears to be moving toward more specialized and sophisticated models that offer practical insights into brain function, neural behavior, and potential interventions for neurological conditions.

## 4 Future directions

The current study lays the groundwork for a multitude of promising avenues in the synergistic fields of deep learning and neuroscience. Given the rapid advancements in both domains, it is imperative that future research endeavors are strategically directed toward harnessing their combined potential to unravel the complexities of the brain and address neurological disorders more effectively. An essential step forward involves expanding the scope of bibliometric analyses to include a broader spectrum of bibliographic databases. Beyond the Web of Science Core Collection, incorporating databases such as PubMed, Scopus, and Google Scholar would provide a more comprehensive overview of the literature. This expansion is crucial for capturing a wider array of research activities, including those published in emerging or specialized journals that may offer novel insights or methodologies at the intersection of deep learning and neuroscience. The field’s rapid evolution calls for an acute focus on integrating the latest deep learning technologies. Emerging frameworks like Generative Adversarial Networks (GANs), Reinforcement Learning (RL), and Transformer models hold significant promise for advancing our understanding of neural mechanisms and brain disorders. Future research should prioritize these cutting-edge technologies, exploring their potential to open new vistas in neuroscience research, from enhancing diagnostic classifications to uncovering novel therapeutic targets. Moreover, complementing quantitative bibliometric analyses with qualitative assessments offers a pathway to deeper insights. Incorporating content analysis of key publications and expert interviews could elucidate the nuances of how deep learning impacts neuroscience. Such a qualitative approach would provide a richer understanding of methodological advancements and theoretical contributions, spotlighting the intricate interplay between computational models and biological insights. Interdisciplinary collaborations stand as a cornerstone for future progress. Bridging the gap between computer scientists, neuroscientists, and clinicians can catalyze the development of innovative solutions to complex neurological challenges. This collaborative approach not only facilitates the translation of computational models into practical clinical applications but also enriches our theoretical understanding of the brain through the lens of deep learning. Another vital direction for future research is the application of deep learning technologies to neurological disorders and mental health. Developing predictive models for early diagnosis, crafting personalized treatment strategies, and deepening our understanding of the neural bases of these conditions can significantly impact public health. Such applications necessitate careful consideration of ethical issues and data privacy, underscoring the need for comprehensive frameworks and guidelines for the ethical use of artificial intelligence in neuroscientific research. Lastly, exploring the analogies between artificial neural networks and brain functions could yield ground-breaking insights into both domains. Future investigations should delve into how deep learning models can mimic cognitive processes and identify neuroscientific principles that can inform the development of more efficient and robust AI systems. This reciprocal learning process not only advances our understanding of the brain but also propels the field of artificial intelligence forward.

The present bibliometric analysis has illuminated a path forward, outlining critical directions for future research that promise to deepen our understanding of the brain, address neurological challenges, and explore the full potential of deep learning in neuroscience. By embracing these directions, the scientific community can foster a rich, interdisciplinary dialog that propels both fields toward unprecedented discoveries and innovations.

## 5 Conclusion and limitations

In our bibliometric analysis, we have documented the evolution and burgeoning integration of deep learning technologies within the field of neuroscience from 2012 to 2023. The results of this study reveal a marked and consistent increase in research output, highlighting the scientific community’s escalating interest and investment in leveraging deep learning to unravel the intricacies of neural mechanisms and tackle neurological challenges. Central to our findings is the pivotal role of classification algorithms, models, and neural networks, which have emerged as foundational pillars in interpreting complex neural data, simulating brain functions, and facilitating the translation of theoretical insights into practical applications such as diagnostics and therapeutic interventions. Furthermore, our analysis uncovers a rich tapestry of research themes that reflect both the diversity and unity of efforts aimed at understanding and manipulating the brain’s function through deep learning. Notably, the identification of emerging research hotspots and the detailed thematic evolution map illustrate the field’s dynamic progression, adaptability to technological advancements, and its ongoing maturation. These insights underscore the critical impact of deep learning on neuroscience, signifying a promising trajectory toward ground-breaking discoveries and innovations in understanding the cerebral code. This study not only highlights the significant contributions and potential of deep learning in neuroscience but also serves as a strategic roadmap, encouraging further exploration, interdisciplinary collaborations, and the harnessing of novel computational techniques to advance our knowledge of the brain.

This study makes a pivotal contribution to the interdisciplinary field of deep learning and neuroscience by offering the first comprehensive bibliometric analysis that charts the evolution, trends, and integration of deep learning technologies within neuroscience research over a crucial period from 2012 to 2023. Through a meticulous examination of 421 articles published during this time, the study not only documents the rapid increase in research output but also identifies the central role of classification algorithms, models, and neural networks in advancing our understanding of the brain. By analyzing keyword frequencies, thematic maps, and trends, it provides a macroscopic view of the dynamic landscape of research, highlighting emerging hotspots and the evolution of key themes. This contribution is significant as it offers a detailed overview of the field’s trajectory, facilitating a deeper understanding of how deep learning has become an indispensable tool in neuroscience. Furthermore, the study acts as a strategic roadmap for future research, pointing out areas that require further exploration and suggesting potential interdisciplinary collaborations. Thus, it not only illuminates the past and present state of the art but also guides the scientific community toward promising directions for innovative discoveries and applications in decoding the complexities of the cerebral code.

Acknowledging the limitations of our bibliometric analysis is crucial for a nuanced interpretation of its findings and for guiding future research endeavors. Primarily, our reliance on the Web of Science (WoS) Core Collection, while ensuring access to high-quality publications, may inadvertently narrow the scope of literature reviewed, potentially omitting pertinent studies published in journals not indexed within this database. Such a limitation underscores the challenge of capturing the entirety of research at the deep learning and neuroscience nexus. Additionally, the inherently retrospective nature of bibliometric analyses introduces a temporal lag, rendering our study less reflective of the latest advancements in deep learning technologies, a rapidly evolving field where novel applications and methodological breakthroughs are continuously emerging. This analysis, focused on quantitative metrics and keyword-based thematic exploration, may also not fully encompass the qualitative depth of research impacts, the intricacies of methodological innovation, or the subtle interplays between computational and biological insights, which are essential aspects of the field’s progression. Moreover, the dependency on specific keywords for thematic analysis may not adequately capture emerging themes or the full diversity of interdisciplinary research, given the variability in terminology. These limitations highlight the importance of extending future bibliometric analyses to include a wider array of databases, incorporating more recent publications, and possibly integrating qualitative assessments to provide a more comprehensive and up-to-date overview of the field’s dynamic landscape.

## Data Availability

Publicly available datasets were analyzed in this study. This data can be found at: https://www.webofscience.com.
